# Hostile Environments: Modifying Surfaces to Block Microbial Adhesion and Biofilm Formation

**DOI:** 10.3390/biom15060754

**Published:** 2025-05-23

**Authors:** Derek Wilkinson, Libuše Váchová, Zdena Palková

**Affiliations:** 1 Faculty of Science, Charles University, BIOCEV, 128 00 Prague, Czech Republic; vachova@biomed.cas.cz; 2 Institute of Microbiology of the Czech Academy of Sciences, BIOCEV, 142 00 Prague, Czech Republic

**Keywords:** microbial biofilms, modified surfaces, yeast and bacterial adhesion, antimicrobial modifications of materials

## Abstract

Since the first observations of biofilm formation by microorganisms on various surfaces more than 50 years ago, it has been shown that most “unicellular” microorganisms prefer to grow in multicellular communities that often adhere to surfaces. The microbes in these communities adhere to each other, produce an extracellular matrix (ECM) that protects them from drugs, toxins and the host’s immune system, and they coordinate their development and differentiate into different forms via signaling molecules and nutrient gradients. Biofilms are a serious problem in industry, agriculture, the marine environment and human and animal health. Many researchers are therefore investigating ways to disrupt biofilm formation by killing microbes or disrupting adhesion to a surface, quorum sensing or ECM production. This review provides an overview of approaches to altering various surfaces through physical, chemical or biological modifications to reduce/prevent microbial cell adhesion and biofilm development and maintenance. It also discusses the advantages and disadvantages of each approach and the challenges faced by researchers in this field.

## 1. Introduction

### 1.1. Biofilms: Discovery, Importance and Formation

Bill Costerton pioneered biofilm research, having observed cell attachment and “glycocalyx” (extracellular matrix―ECM) production in bacteria from the rumen of cows [1,2,3,4]. He later identified similar bacterial lifestyles on rocks in streams, in urinary catheters, within lungs, and in many settings in agriculture, industry and medicine [4,5,6,7,8,9,10,11,12]. It is now widely accepted that bacteria, fungi and other microorganisms prefer to grow in communities attached to biotic or abiotic surfaces embedded in the ECM and protected from environmental challenges [13,14,15]. Biofilms have beneficial roles, including in probiotics, food fermentation, curing and ripening, the production of chemicals, wastewater treatment, stimulation of plant growth, bioprotection, biofertilizers, bioremediation, anti-corrosion treatment and microbial fuel cells [16,17,18,19]. Biofilms of “friendly” microorganisms also colonize human (and other animal) skin and mucous membranes, protecting against colonization by pathogens, facilitating digestion and producing vitamins [20,21]. Altering the balance of gut microbes causes obesity, diabetes, cancer, nerve pathologies, liver disease, allergies, inflammation and behavioral changes [22,23]. On the other hand, biofilms can be the cause of serious infections in medicine and contamination in industry.

**Figure 1:**
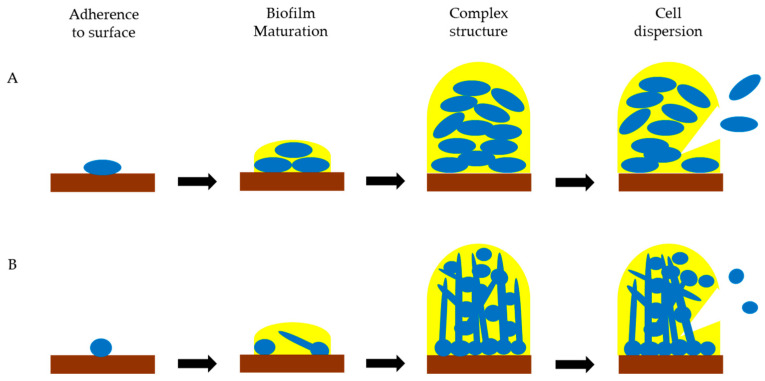
Stages in biofilm development by bacteria (**A**) and the yeast *C. albicans* (**B**). Brown: surface, Blue: microbial cells, Yellow: ECM.

Biofilm formation by bacteria and yeasts (Figure 1) [24,25,26,27] begins with adhesion to a biotic or abiotic surface via charge or hydrophobic interactions and van der Waals forces, etc., but later, adhesion proteins (adhesins) become dominant. Adhesins promote biofilm formation, and strains that lack adhesins are often less virulent. Examples of fungal adhesins include ALS (agglutinin-like sequence) and HWP (hyphal wall protein) adhesins of *Candida albicans* that adhere to host ECMs and transglutaminases and Epa1 (epithelial adhesin) of *Nakaseomyces glabratus* that adhere to host cell carbohydrates. Some adhesins are only expressed by yeast or filamentous cell forms, and some proteins with other functions also act as adhesins, e.g., glyceraldehyde-3-phosphate dehydrogenase (GAPDH). Bacteria have a negatively charged cell surface and tend to adhere to positively charged surfaces. The fimbriae and pili of some bacteria participate in attachment, and the *Escherichia coli* fimbrial adhesin FimH plays a role in attachment to host mannosides, laminin, fibronectin and plasminogen. The *Staphylococcus aureus* adhesin FnBPA (fibronectin-binding protein A) adheres to fibronectin and fibrinogen in the host as well as to FnBPA on other *S. aureus* cells. The adsorption of proteins to surfaces increases the range of moieties to which microorganisms can adhere. Examples include the adsorption of fibronectin and vitronectin to the surface of biliary stents [28] and serum proteins such as collagen, fibronectin and immunoglobulin G to titanium surfaces [29].

In the next stage (biofilm maturation, Figure 1), the ECM (also known as glycocalyx or extracellular polymeric substances) is secreted by adhered cells and protects microbes from dehydration, toxins, immune responses and antimicrobial drugs. The ECM consists mainly of exopolysaccharides such as cellulose, alginate, levan type I and II (both fructose polymers) and Psl in various bacteria, and α-mannan and β-1,6-glucan in fungi [30,31]. Other components of the ECM include proteins, lipids and nucleic acids. The ECM is also a reservoir of water and nutrients. Microbes adhere to one another and secrete signaling molecules. Following biofilm maturation, complex structure develops (Figure 1) with water channels and gradients of oxygen, nutrients, waste products, pH and signaling molecules, and some cells may become persister cells―cells that are tolerant towards antimicrobials and environmental change. The next step is biofilm dispersion, when microbes secrete enzymes and surfactants that break up the ECM, allowing cells to return to a single-celled lifestyle, spread from the biofilm and potentially colonize other surfaces. The stages of biofilm development were first observed in bacteria (Figure 1A). Many *C. albicans* cells in biofilms switch to hyphal growth during biofilm maturation [32], but dispersion involves cells in the yeast phase (Figure 1B).

### 1.2. Biofilms in Medicine and Industry

In medical care, 110,000 U.S. patients with an orthopedic implant suffer a nosocomial infection due to biofilm formation on the implant, and implant-associated infections approach 1,000,000 per annum [33]. Drug tolerance (phenotypic resistance) can be 5000 times higher for bacteria in biofilms [34]. The yeast genus *Candida* has been detected in 25% of urinary tract infections and 10% of bloodstream infections [35]. *C. albicans* produces persister cells in biofilms that are phenotypically tolerant to antifungal drugs. The production of persisters depends on cell adherence and surface material, and the number of persister cells remains stable as the biofilm grows [36]. Further, 40% of cases where a catheter becomes colonized by yeast result in blood stream infections, leading to 40% mortality [37]. Indwelling catheters adsorb proteins, providing an interface for microbial attachment and leading to biofilm formation [38]. The complex architecture and extracellular matrix of biofilms block drug diffusion, the upregulation of efflux pumps excludes drugs from cells, and metabolic differences (e.g., dormancy) counteract drug activity [34,39,40].

Biofilms are a problem in industry and form on fluid pipelines, membranes, work surfaces, storage bins, etc., and can be lethal if they include pathogens such as *Bacillus cereus* (which causes diarrhea or vomiting), *Listeria monocytogenes* (which causes listeriosis) and *Salmonella enterica* (which causes salmonellosis) [41]. Drinking water pipes are contaminated by biofilms that alter the smell, flavor and appearance of water, cause corrosion and may include pathogens such as *Salmonella typhimurium* (which causes enteric or typhoid fever), *Legionella pneumophila* (which causes legionnaire’s disease) and *Helicobacter pylori* (which causes stomach ulcers), all of which are more resistant to disinfectants when growing as biofilms [42].

This review covers different methods of modifying surfaces to reduce adhesion and/or biofilm formation by microorganisms (Appendix A). This includes physical modifications to surfaces that affect the roughness and wettability. It also includes chemical modifications that influence the hydrophilicity, charge or electrical conductivity of the surface or that use a positively charged nitrogen atom to attack microbial membranes, proteins and nucleic acids. In addition, it covers the attachment or embedding of an antimicrobial peptide or antibiotic that has antifungal or antibacterial properties. Finally, this review discusses some of the newer approaches to anti-biofilm surface modification.

## 2. How to Combat Biofilms

### 2.1. When to Combat Biofilms

Once biofilms produce an ECM, they are difficult to treat, since the ECM protects microbial cells from many antifungal or antibacterial drugs, phagocytosis and neutrophil extracellular traps (NETs) [43,44,45]. Some studies use enzymes or bacteriophages to disrupt the ECM [46,47], but bacteria have defenses against phage infection and such approaches are not as effective as hoped, hough phage treatment has been combined with antibacterial drugs, disinfectants or nanoparticles [48,49]. Furthermore, such treatments may disperse cells to seed new biofilms, and dispersed cells can be highly virulent [50]. For all these reasons, many treatments in use today aim to block the first step of biofilm formation―microbial attachment to the surface [51]. This is also important when dealing with *C. albicans*, which only forms drug-tolerant persister cells after adherence to a surface [36].

### 2.2. How to Prevent Cell Adhesion, the First Step in Biofilm Formation

The characteristics of surfaces determine the likelihood and rate of biofilm formation [52]. The hydrophilicity/wettability of a surface is influenced by surface moieties, and some cells are more likely to adhere to more wettable surfaces. Carboxyl and amino groups participate in cell and protein adsorption, and adsorbed proteins provide chemical groups to which microbes can attach [53]. However, microbes respond differently to a range of surface properties. *S. aureus* favors hydrophilic surfaces, while *S. epidermidis* prefers hydrophobic surfaces. *C. albicans* adherence and biofilm formation was reduced when polyethylene terephthalate (PET) was modified to be more hydrophilic or more cationic [54]. Greater hydrophilicity, achieved via oxygen glow discharge (treatment with ionized gas) on polyvinyl chloride, reduces *Pseudomonas aeruginosa* adhesion by 70% [55]. Plasma treatment (bombardment with a mixture of ions and electrons) also increased surface roughness, reducing microbial adherence. The size of surface hills, valleys, etc., affects microbial adhesion [52]. Where valley size and microbe size are similar, adherence is more likely, while where valleys are smaller, adherence is less likely.

The surfaces of catheters, medical implants, pipes, etc., may be modified to prevent biofilm formation by killing microbes or inhibiting attachment [51]. Countering adhesion and biofilm formation via surface modification has been reviewed by several groups [52,56,57,58,59,60,61]. There are ways [59,61] to prevent or reduce adhesion, halt the growth of microorganisms or kill them with techniques that use physical, chemical or biological effects (Table 1).

## 3. Physical Modifications of Surfaces

**Figure 2:**
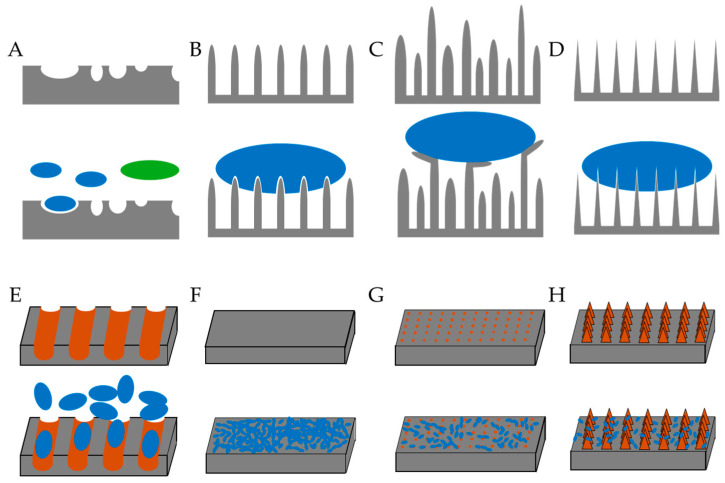
Natural and manufactured antibacterial surface modifications. (**A**) Surface roughening, (**B**) nanopillars on the surface of cicada wings, (**C**) nanopillars on the surface of dragonfly wings, (**D**) manufactured nanopillars, (**E**) troughs created in a surface, (**F**) biofilm formation on unmodified surface, (**G**) biofilm formation on surface on which nanodots have been deposited, and (**H**) biofilm formation on surface on which more complex spires have been deposited. Gray: surface, Orange: troughs made in surface or structures deposited on surface, Blue/Green: microorganisms.

Physical techniques (Table 1) may involve using lasers to modify surfaces via melting, alloying or surface structuring to yield structures with a small surface area and changed hydrophobicity [57,62]. Roughening a surface, to produce “valleys” that are smaller than a particular microbe, reduces colonization [63] (Figure 2A). Lithography, X-rays, electrons, charged particles, etc., deposit specifically sized and shaped structures onto a surface, reducing adhesion and biofilm formation, while plasma treatment attaches groups to surfaces to change their properties [57]. Such changes may be random or ordered [60]. Ordered changes are more demanding and are inspired by natural structures such as the lotus leaf surface, animal wings, scales, etc. In nature, cicada and dragonfly wings are covered in “nanopillars”. The regularly sized nanopillars on cicada wings (Figure 2B) exert stress, causing deformation of the Gram-negative bacterial membrane between pillars, disrupting the membrane and killing the bacterium, but due to their more robust cell membrane, Gram-positive bacteria are protected against this strategy [60]. The surface of dragonfly wings kills both Gram-positive and Gram-negative bacteria as they have differently sized nanopillars (Figure 2C), and the taller pillars bend as bacteria adhere, pulling on the membrane and exerting pressure sufficient to disrupt membranes of both types of bacteria.

Unfortunately, surface modifications that reduce pathogen adherence may also reduce host cell adherence during tissue regeneration or reduce the adherence of commensal cells that control pathogens [72]. The choice of material for medical implants, etc., is also important. Steel is easier to work with than titanium, but titanium has greater strength, is not as dense, is protected from corrosion by its layer of titanium oxide and supports new bone formation [89]. Gold nanopillars have been produced, which combat microbial adhesion but are also biocompatible [90]. Each method of surface modification has advantages and disadvantages, including complicated manufacturing procedures, the need to heat the material to a high temperature, long production times and expensive machinery and processes [62,91]. Several teams have tested the deposition of metal onto the surface of a polymer followed by treatment that creates patterns in the metal surface. Fu et al. [92] bombarded metal with ionized gas plasma to deposit a thin layer of gold or silver on polystyrene by sputtering and then heated it to shrink the coating, creating wrinkles less than 1 μm apart. Another team used sputtering to deposit gold and silver on the surface and heating to increase roughness and hydrophobicity and then made a polydimethyl siloxane mold that they used to cast polystyrene, polycarbonate and polyethylene surfaces [93]. The team showed that *E. coli* adherence was greatly reduced by structuring the surface, particularly for polycarbonate and polyethylene.

Examples of the use of surface modifications by various physical methods are presented below and in Table 1.

### 3.1. Laser Treatment

Using a laser to roughen a polyethylene surface and introduce 0.6–2 μm “valleys” decreased wettability and reduced colonization by *E. coli* but not by *S. aureus* [63]. Smaller (0.5–1 μm) *S. aureus* cells may fit into the “valleys”, while larger (>1 μm) *E. coli* cells do not (Figure 2A). It has also been shown that covering titanium implants with a layer of anatase (a titanium oxide mineral) reduces the width of surface “valleys” from 5 μm to 0.4 μm, reducing the adherence of *E. coli* by up to 50% [94]. Laser modification of surfaces is also effective against fungi, and altering stainless steel using laser-induced periodic surface structures (LIPSSs) introduced 630 nm patterning, resulting in a reduction in biofilm formation by a wild (biofilm-forming) strain of *Saccharomyces cerevisiae* [62]. Laser treatment is effective, but the equipment is extremely expensive (Table 2).

### 3.2. Lithography

**Figure 3:**
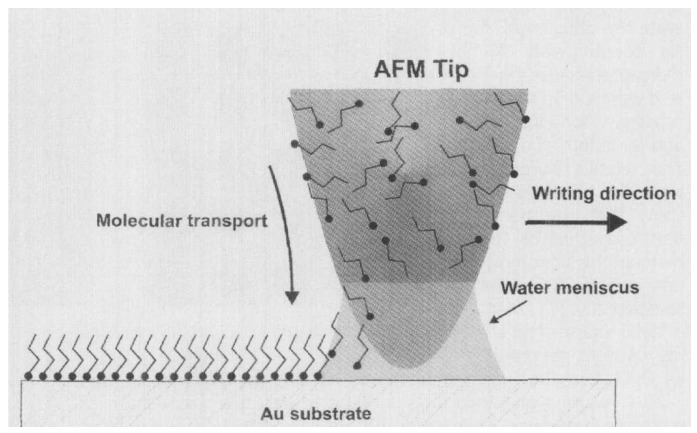
Dip pen nanolithography. Reproduced from [98] with permission from *Science*.

**Figure 4:**
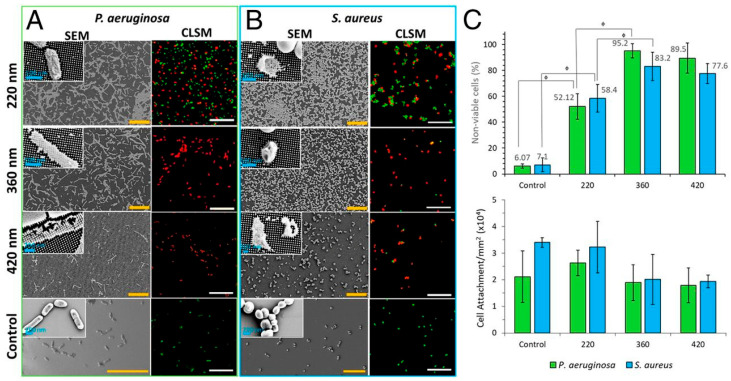
Effect of nanopillar size on bacterial viability. Mixture of Syto 9 and propidium iodide used for live/dead staining. Syto 9 (green) crosses the membrane of live and dead cells, staining nucleic acids, etc., while propidium iodide (red) enters only dead cells but binds nucleic acids with greater affinity than Syto 9. (**A**) *P. aeruginosa*, (**B**) *S. aureus*, (**C**) non-viable (upper bar chart) and attached (lower bar chart) cells. Scale bars: 20 μm. Statistical significance is denoted by ɸ*p* < 0.05. Reproduced from [95] with permission from PNAS.

Lithography is a well-known method used in printing [96]. However, dip pen nanolithography (DPN―using an atomic force microscope tip to apply molecular “ink” under a meniscus of water) can also be used to print nanoscale structures onto surfaces (Figure 3) and reduce microbial adherence [97,98]. DPN was used [64] to apply patterns of dots on silicon/gold alloy. A synthetic polymer, polydimethylsiloxane, was used to create a mold of the resulting surface and then applied to silica sol on stainless steel to yield a similar patterned surface, consisting of silica dots on stainless steel. *Streptococcus mutans* adherence on silicon-patterned stainless steel was lower than that on diamond-paste-polished or silica-dip-coated stainless steel. Silica nanopillars of different heights were deposited on a surface by UV immersion lithography (Table 1) (Figure 4), and live/dead staining was used to identify viable (green) and non-viable (red) cells. The greater the nanopillar height, the greater the reduction in bacterial viability in *P. aeruginosa* (A) and *S. aureus* (B) [95].

### 3.3. Plasma Treatment

**Figure 5:**
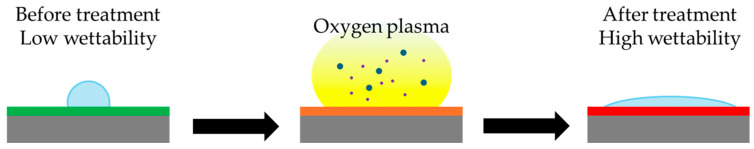
Effect of plasma treatment on wettability. Green: untreated surface, orange: plasma-bombarded surface, red: treated surface, blue: water drop. Adapted from [101].

Plasma treatment (bombardment with electrons, ions, atoms, molecules, etc.) can modify polyvinyl chloride, polyethylene terephthalate and polyurethane surfaces, increasing the wettability (Figure 5) and reducing the adhesion of *P. aeruginosa*, *S. aureus*, *E. coli* and *S. epidermis* [57]. Plasma treatment turns molecules into reactive species that attach to a surface, and adding carboxyl, amino or alkyl groups influences the surface’s chemical properties [52]. Glow discharge uses incompletely ionized gas, such as carbon dioxide and ethylene, to modify the roughness, charge density and free energy of adhesion of a polymer surface and showed that while the first two had no effect, the free energy of adhesion was proportional to microbial adhesivity [74]. A silicon/boron wafer was etched via reactive ion etching (RIE) using sulfur hexafluoride and oxygen to produce nanopillars similar to those on the wings of cicadas and dragonflies [65]. This etched silicon material (black silicon―bSi) killed 4.3 × 10^5^ *P. aeruginosa*, 4.5 × 10^5^ *S. aureus* and 1.4 × 10^5^ *B. subtilis* cells per cm^2^ per minute. It also (like dragonfly wings) killed *B. subtilis* spores. Plasma treatment of stainless-steel enhanced oxygen content, wettability and nano-structuring, yielding a superhydrophilic surface and reducing adhesion by *E. coli* and *S. aureus* [99]. When titanium was subject to plasma electrolytic oxidation with or without silver or zinc, bacterial viability was greatly reduced on the silver- or zinc-spiked surfaces [100] Plasma treatment can combat microbial adhesion by enhancing wettability (Figure 5) [101].

### 3.4. Electron Beam

**Figure 6:**
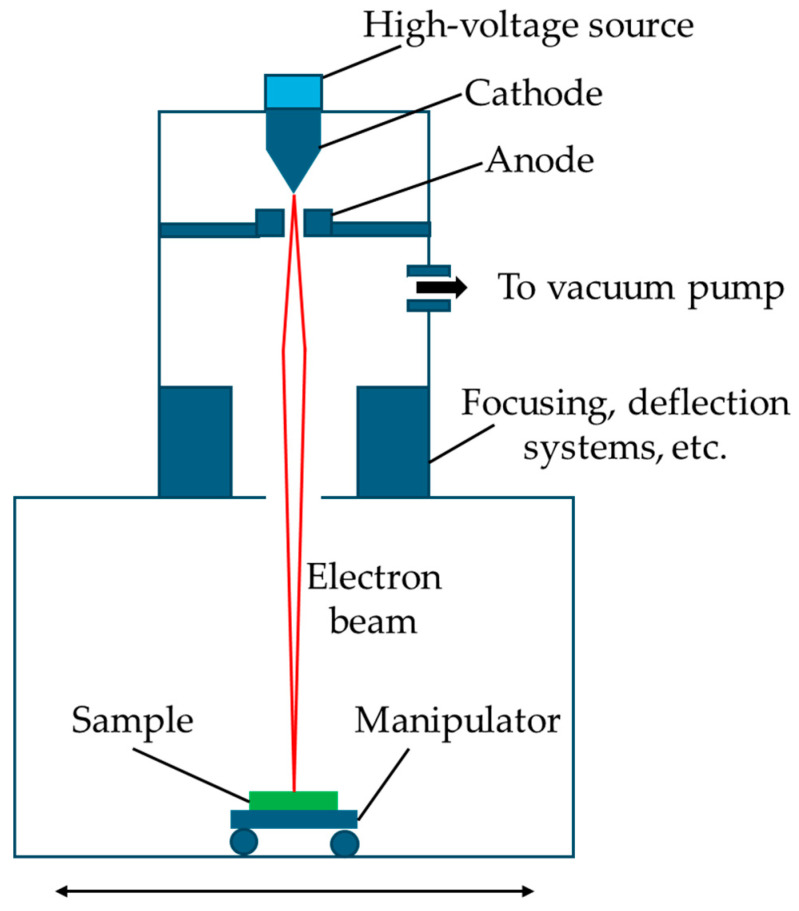
Electron beam treatment of surfaces. Adapted from [102].

Heating titanium pellets with an electron beam (EB) to coat titanium foil (Figure 6) [102] and produce a rough surface resulted in significantly reduced biofilm formation by *S. aureus*, *S. epidermidis* and *P. aeruginosa* [66], and the effects of further surface treatment of EB-coated titanium pellets on adherence were tested [67]. Acid etching of the pellets increased *C. albicans* adherence, while machining and sandblasting decreased adherence. All three surface treatments reduced *S. aureus* adhesion, while all increased *P. aeruginosa* adhesion. These results demonstrate that the effects of surface roughness may be species-specific.

### 3.5. Physical Vapor Deposition

Another method, physical vapor deposition, was used to create gold surfaces with various patterns, including a grainy pattern and channels roughly the size of the bacteria being tested [103]. *P. fluorescens* were largely found aligned in the channels, and cells were shorter and differently shaped. The channels acted as traps, preventing bacteria from reaching the surface. The channels interfered with colonization, swarming and twitching. The channels also affected cell aggregation, preventing the formation of large bacterial aggregates [68].

## 4. Chemical Modifications of Surfaces

**Figure 7:**
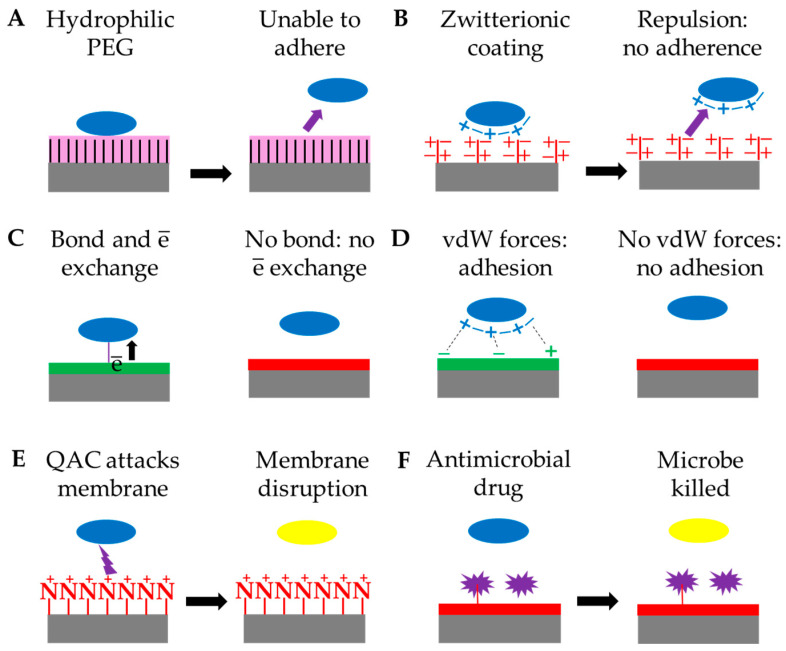
Chemical modification of surfaces. Gray: medical/industrial material, green: unmodified surface, red: modified surface, pink: water, purple: antimicrobial compound or effect, blue: live microorganism, yellow: dead microorganism. (**A**) Microbes tend to favor hydrophobic surfaces and hydrophilic PEG becomes saturated with water, reducing adhesion, (**B**) zwitterionic coatings have equal numbers of positive and negative charges that repel charges on microbes, (**C**) adhesion to some surfaces involves electron exchange between microbes and surfaces and coatings may be used to reduce electron exchange and thus adhesion, (**D**) Surfaces may be modified to reduce the availability of charged groups that participate in adhesion, based on van der Waals forces, (**E**) QACs have positively-charged nitrogen atoms that are attracted to negatively-charged microbial membranes, penetrate and disrupt the membranes, (**F**) antimicrobial drugs may be attached to or embedded in a surface, killing microbes or inhibiting their growth.

The chemical modification of surfaces involves coating the surface (Figure 7) or attaching groups with specific chemical properties. Some antimicrobial polymers, such as polyethylene glycol (PEG) and zwitterionic polymers, are passive―blocking the adsorption of microbial proteins (Table 1), while others, such as quaternary ammonium compounds (QACs), are active―attacking and killing the microorganisms [61]. Major concerns of chemical modification of surfaces include toxicity, bioaccumulation, biodegradation and the specificity of the effect. Longer QAC chains and ring structures increase toxicity towards other organisms. Copper-based and pesticide-based paints are effective at controlling biofouling, but they are toxic and remain in the environment [104].

Various techniques can be used for attaching moieties [59], including the adsorption and grafting of specific groups. Silver, in the form of coatings, mixed-metal surfaces or nanoparticles kill microorganisms by a combination of silver toxicity, silver REDOX reactions and (on a silver/palladium surface) electric fields [105]. Biofilm formation on different dental cements may depend on differences in conductivity and the exchange of electrons between bacteria and surfaces during adherence [106]. Antimicrobial drugs may also be attached to surfaces or embedded within a semi-porous material, allowing the drug to diffuse out at a rate which ensures microbial killing but avoids toxicity against the patient’s own cells [81]. The choice of drug is crucial. For example, non-*albicans Candida* species have evolved greater resistance to many drugs in recent years and are increasingly being isolated from candidiasis patients. In addition, antifungal drugs can have severe side-effects and can damage host organs, but low doses can lead to drug resistance in target microorganisms [107]. For these reasons, the release of drugs from surface coatings must be carefully regulated or the drug must be tethered to the surface.

Some chemical modifications of materials increase or decrease hydrophobicity [61], but some microbes, such as *C. albicans*, can exist in two forms―one with higher cell surface hydrophobicity and one with lower cell surface hydrophobicity, and the former is regarded as more virulent [108,109]. *C. albicans* yeast-shaped cells are more hydrophilic, while hyphae are more hydrophobic, and some experiments find no effect of surface wettability on *C. albicans* adherence [81].

Examples of the use of surface chemical modifications are presented below and in Table 1.

### 4.1. Passive Chemical Modification

#### 4.1.1. Polyethylene Glycol (PEG)

One passive chemical modification technique (Figure 7A and Table 1) involves attaching PEG to the surface of a material to form a brush-like covering which absorbs water. This was shown to reduce biofilm formation by *S. epidermidis*, *S. aureus* and *S. salivaris* by at least 94% and that by *C. aeruginosa*, *C. albicans* and *C. tropicalis* by 80% [69]. Attaching PEG reduces cell adherence by reducing protein adsorption [53], but protein adsorption and cell adhesion are affected by hydrophilicity, the absence of charges and chain length, mobility and density [110]. PEG is safe and effective but attached groups can trigger immune responses and the chemical bond can be oxidized [61] (Table 3).

#### 4.1.2. Zwitterions

**Figure 8:**
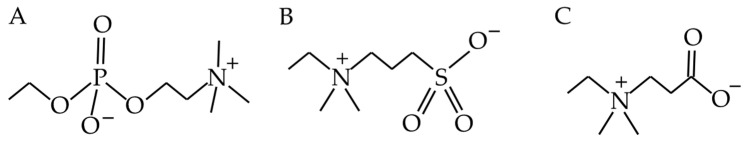
Structures of three common zwitterions phosphorylcholine (**A**), sulfobetaine (**B**) and carboxybetaine (**C**). Adapted from [111].

Polymers, such as carboxy betaine, phosphorylcholine and sulfobetaine, are zwitterionic (Figure 7B and Table 1), with similar numbers of positive and negative charges, increasing hydrophilicity and decreasing the adherence of *E. coli* and *S. aureus* [61]. Three older antimicrobial zwitterionic compounds [111] are phosphorylcholine (Figure 8A), sulfobetaine (Figure 8B) and carboxybetaine (Figure 8C). Chains of alternate basic lysine and acidic glutamate residues formed a brush-like layer on the surface of hydrophobic polystyrene and caused a 70% reduction in adsorption of extracellular material from *P. aeruginosa*, an 80% reduction in *P. aeruginosa* cell adhesion and a reduction in biofilm thickness [70]. Masotti et al. investigated the effect of hydrophobicity and hydrophilicity of surfaces on *C. albicans* adherence since there were conflicting accounts. They grafted zwitterionic (hydrophilic) or fluorinated (hydrophobic) copolymers onto glass slides and tested for *C. albicans* adherence, using glass as a negative control (glass resists *C. albicans* adherence). The fluorinated copolymer performed almost as well as bare glass, but adherence was greatly increased on zwitterionic copolymers [71]. Zwitterions are generally biocompatible, with low toxicity and immunogenicity [111].

#### 4.1.3. Other Modifications of Polymers to Change Hydrophobicity or Charge

Several polymers were directly modified (Table 1) to change their chemical properties. Polyether–urethane copolymers were modified by the addition of polyethylene oxide, fluorocarbon or silicone, while polyethylene terephthalate was modified with different surface-modifying end groups (SMEs) to render them hydrophobic, hydrophilic, anionic or cationic. *C. albicans* biofilm formation was significantly increased when polyethylene terephthalate was modified with cationic or hydrophilic SMEs, while the addition of polyethylene oxide to polyether urethane reduced *C. albicans* biofilm development significantly [37].

#### 4.1.4. Electrical Conductivity

Substances used by dentists may be made of metals, ceramics or polymers [72] and include cements, resins and implants. Mixing zirconia (a zirconium oxide ceramic) with an acrylic polymer significantly lowered mixed biofilm formation by *Streptococcus sanguinis*, *Porphyromonas gingivalis* and *Fusobacterium nucleatum*, possibly via altered electrical conductivity (Figure 7C and Table 1), and biofilm formation was still lower on the acrylic polymer itself [73].

#### 4.1.5. Superhydrophobic and Superhydrophilic Surfaces

Superhydrophobic surfaces have extremely low wettability and include siloxane and fluorosiloxane (TCFS) deposited on silicon or boron-doped titanium surfaces using a plasma jet as well as hexamethyldisiloxane (HMDSO) and Teflon. The superhydrophobic surfaces had nano-patterned surfaces. Bovine serum albumin and fibrinogen adsorption were greatest on the hydrophobic HMDSO, whereas hydrophobic TCFS had the lowest adsorption of both proteins. *S. aureus* adherence was much lower on superhydrophobic TCFS-coated titanium than on uncoated titanium. Van der Waals forces may be reduced on hydrophobic TCFS-covered silicon (Figure 7D and Table 1), leading to reduced adsorption [58,118]. Problems associated with creating such superhydrophobic surfaces include the need for expensive equipment and a high energy requirement, pollution, toxicity and poor performance in some cases [113].

#### 4.1.6. Adding Charge to a Surface

In one study, the pretreatment of polyurethane sheets with glow discharge and oxygen was followed by plasma grafting to attach acrylic acid, butyl acrylate or methyl vinyl to the polymer surface, and some of the polyurethane/acrylic acid polymer was treated with silver nitrate. It was found that the smoothness or hydrophobicity of the surface did not influence the level of bacterial colonization, but a negative charge on the polymer surface (Table 1) led to reduced adherence by *S. epidermidis*. However, adherence was independent of charge density. Silver ions at the surface of the silver nitrate-treated polymer killed all adhered bacteria within 48 h [74].

#### 4.1.7. Poly Sodium Sulfonate

**Figure 9:**
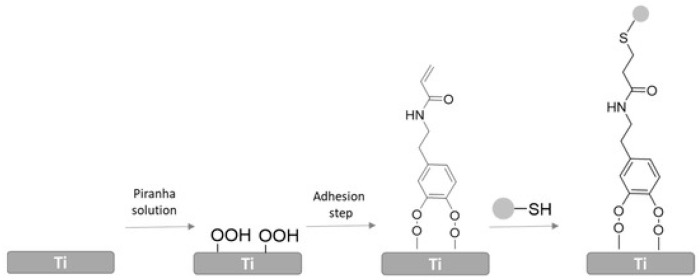
Attachment of polyNaSS to titanium via catechol. Reproduced from [122] https://creativecommons.org/licenses/by/4.0/ (accessed on 20 April 2025).

When sodium polysulfonate (polyNaSS) is grafted onto a surface, it changes the conformation of proteins that adsorb to the surface, preventing the binding of microorganisms to the adsorbed proteins. [119]. Sulfuric acid and hydrogen peroxide react with titanium to yield hydroxide and peroxide. Heating this with sodium styrene sulfonate yields radicals that induce the grafting of polyNaSS to the surface [120]. Another method that generates polymerization-triggering radicals involves the use of UV light [119]. Grafting polyNaSS to titanium surfaces reduced MRSA *S. aureus* adherence by 80 or 90% without affecting osteoblast adhesion [75,121]. Another anchoring method involves attaching catechol (1,2-dihydroxybenzene) or a catechol derivative to the surface and then attaching polyNaSS [59,122] (Figure 9). Grafting polyNaSS onto polyaryletherketone reduces microbial adhesion and enhances hydrophilicity, protein adsorption, bone repair and biocompatibility [114].

### 4.2. Active Chemical Modification

#### 4.2.1. Quaternary Ammonium Compounds (QACs)

QACs consist of one positively charged nitrogen and four alkyl moieties. The ideal alkyl chain length for attacking Gram-positive bacteria is shorter than that for Gram-negative bacteria [61]. QACs are surfactants that induce micelle formation in cell membranes by reducing surface tension, disrupting the membranes and causing cytoplasmic leakage. They react with intracellular proteins and nucleic acids, and they trigger cell lysis by cellular hydrolytic enzymes [123] (Figure 7E and Table 1). *Cellulophaga lytica* is a Gram-negative marine bacterium that forms biofilms [124]. Tethering about 4% QACs to polysiloxane reduced *C. lytica* biofilm formation by half [76]. When painted onto titanium or stainless-steel implants, the coating reduced biofilm formation by *S. aureus* after implantation into sheep [77]. Covering a PVC catheter with N,N-dodecyl, and methyl-polyethylenimine (DMPEI) polymer, makes the surface smoother and raises the hydrophilicity, reducing colonization by *E. coli* by 89%, that by *S. aureus* by 94% and that by *C. albicans* by 87% [78]. Unfortunately, QACs persist in the environment and can trigger allergies, respiratory and reproductive problems and endocrine disruption [112].

#### 4.2.2. Cationic Dendrimers

**Figure 10:**
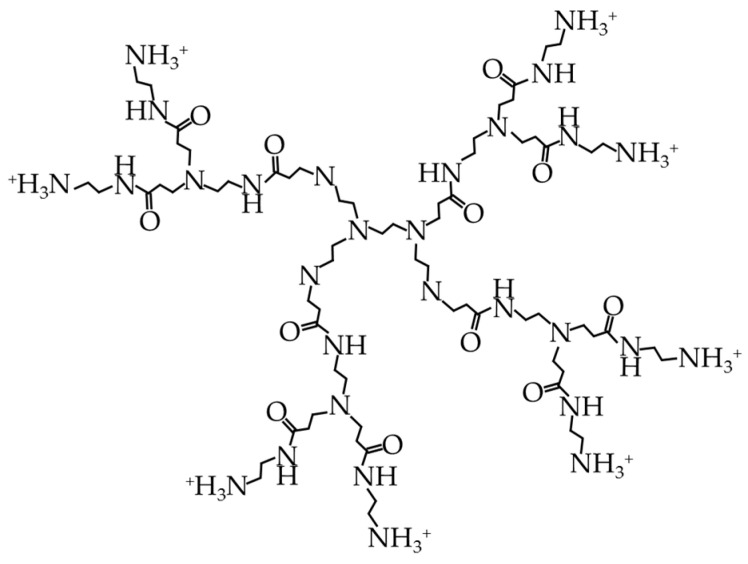
Structure of PAMAM (adapted from [125]).

Cationic dendrimers have a net-positive charge, are active against negatively charged microbial membranes, and have both hydrophilic and hydrophobic portions, enabling them to form pores in microbial membranes, much like some natural antimicrobial peptides (AMPs) [116]―see later. The branching (dendritic) structure and positive charge (Figure 10) can be seen in the structure of poly(amidoamine) (PAMAM) [125]. Dendrimers are effective at low concentrations (Table 3) and resistant to proteolysis (causing high bioavailability), and peptide dendrimers are more effective and biodegradable [117]. Unfortunately, they are cytotoxic and, at a high density, immunogenic. They are expensive and difficult to produce and purify. One method of attaching PAMAM dendrimers to a surface is to plasma-treat a polymer surface in the presence of maleic anhydride and then bind a dendrimer with an amine group to the polymer-bound maleic group [126]. Another group [127] produced self-assembling peptide dendron nanoparticles from modified dendrimer molecules with repeating arginine–proline units. The SPDNs were effective at reducing the viability of *E. coli*, *P. aeruginosa*, *S. typhimurium*, *S. aureus*, *S. epidermidis*, *E. faecalis* and MRSA. A hydrogel containing ketoconazole and PAMAM had a greater effect on *C. albicans* viability than one containing ketoconazole alone, possibly because PAMAM improved the solubility of the antifungal drug [115].

#### 4.2.3. Nanoparticles

Stainless steel is strong and easy to shape, so it was initially used in dental implants. However, microorganisms colonize the area between the implant and the bone, so stainless steel is now used for temporary implants, and cobalt alloys, titanium and polyether ether ketone are used instead [72]. Silver nanoparticles (Table 1) embedded in titanium kill microorganisms and prevent biofilm formation [79]. Electron movement between titanium and silver may generate reactive oxygen species (ROS) that kill microorganisms [128]. A surface may be covered in calcium phosphate using a sol–gel process (successive hydrolysis and condensation), leading to a reduction in adhesion by *S. mutans*, *S. sanguinis* and *Aggregatibacter actinomycetemcomitans* [72]. Nanoparticles that are effective against *C. albicans* include silver, gold, chitosan, iron oxide, copper, zirconium dioxide and titanium dioxide. They act by downregulating ergosterol biosynthesis or drug efflux, ROS production, inhibition of growth, filamentation, enzyme activity and germination, and downregulation of virulence, morphogenesis and biofilm formation genes. Nanoparticles have synergistic effects with other antifungal agents [129]. Nanomaterials have been developed that respond to stimuli such as reduced pH, the presence of particular enzymes and raised levels of hydrogen peroxide or hydrogen sulfide (produced by some microbes) at the infection site [130]. For example, a change in pH could trigger drug release via the protonation/deprotonation of amine groups. “Intelligent nanomaterials” may be designed with bonds or coatings that are disrupted by infection-related enzymes. Cuprous oxide forms Cu_9_S_8_ in the presence of hydrogen sulfide, which is antibacterial and generates ROS that help kill microorganisms. Nanoparticles have also been developed that are sensitive to light or heat, ultrasound, microwaves and magnetism, enabling their functions to be switched on. Effective antimicrobial coatings may be produced using titanium oxide, zinc oxide, copper oxide, magnesium oxide, calcium oxide, cerium oxide and aluminum oxide nanoparticles [131]. Antimicrobial activity was enhanced by embedding a mixture of magnesium oxide and zinc oxide nanoparticles in acrylic latex.

#### 4.2.4. Calcium Phosphate Coatings

Fluoride and zinc ions have antibacterial properties, so titanium alloy discs were coated using a solution of calcium and phosphate ions (Table 1) (producing a calcium phosphate coating), and the solution was spiked with zinc ions, fluoride ions or both [80]. All coatings reduced biofilm formation by *P. gingivalis* compared with uncoated titanium alloy. The zinc-spiked coating had the greatest effect, followed by the other ion-spiked coatings. Zinc enters microbial cells and interferes with ATP biosynthesis, while fluoride affects the enzymes and membranes of bacterial cells.

#### 4.2.5. Toremifene

Surgical implants are often made of titanium, and biofilms can form on the surface. Braem et al. mixed titanium and titanium hydride, which they molded and dehydrogenated to yield discs with pores. They filled the pores with a sol consisting of aqueous silicon dioxide and hydrochloric acid (Table 1). Discs were placed in the wells of a 12-well polystyrene plate, and the wells were heated and crimped to seal them around the discs. The anticancer drug toremifene, which has been shown to possess antimicrobial properties, was placed under the discs. The greater the concentration of drug, the higher the rate of diffusion via sol-filled pores, so the toremifene release rate may be antimicrobial but not cytotoxic. There was a 70% reduction in the metabolism (and therefore mass) of *C. albicans* biofilm within the wells [81]. Another group showed that toremifene blocks the growth and the biofilm formation of *P. gingivalis* and *S. mutans*, actively kills *S. mutans* and acts by disrupting the cell membrane [132].

#### 4.2.6. SPI031

**Figure 11:**
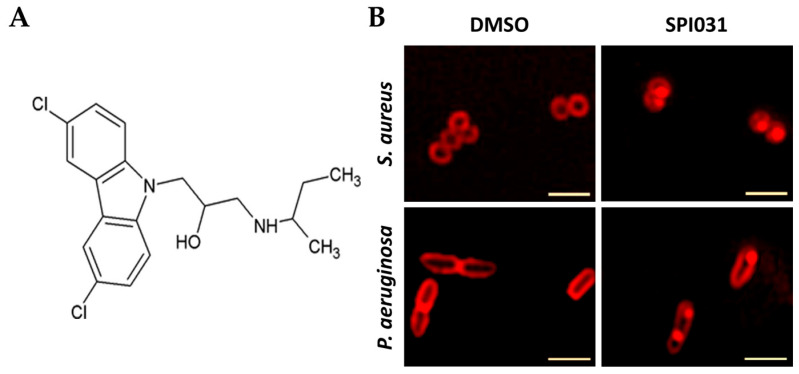
(**A**) Structure of SPI031. (**B**) Effect of SPI031 on cell membranes, visualized by staining with FM 4-64. Reproduced from [133] http://creativecommons.org/licenses/by/4.0/ (accessed on 20 April 2025).

SPI031 is an antibacterial drug (Figure 7F) active against Gram-negative and Gram-positive bacteria. SPI031 was covalently attached to roughened titanium discs using a silane anchor (Table 1). Discs were soaked overnight in a solution of fetal bovine serum to allow adsorption of the protein and then placed in the wells of a 24-well plate, and *S. aureus* or *P. aeruginosa* suspensions were added. SPI031 attachment significantly reduced biofilm formation by both species. The drug was less active against *P. aeruginosa*, possibly because the outer membrane protects the bacterium. SPI031 attachment to titanium discs, implanted into immunosuppressed mice, reduced *S. aureus* biofilm formation on titanium discs by 98% [133]. The structure of SPI031 is shown at Figure 11A, and the result of drug treatment on the membranes of *S. aureus* and *P. aeruginosa* is shown at Figure 11B. The cells were stained with FM 4-64, which stains cellular membranes. There was uniform membrane staining in DMSO-treated controls, but in cells treated with SPI031, there was heterogenous membrane staining, indicating that SPI031 had caused some kind of membrane disruption. The mechanism by which SPI031 attacks the membrane is not known, but some groups are working on variants of the drug with reduced activity against animal cell membranes, and it has been shown that immobilized SPI031 is not cytotoxic against human cells that form bone or blood vessels.

## 5. Biological Modifications of Surfaces

A wide range of antibiotics and antimicrobial peptides have antimicrobial effects (Table 1) and can be incorporated into or attached to coatings on various surfaces to prevent or reduce biofilm formation [59]. The disadvantages of these modifications are that the peptides can be attacked by host hydrolytic enzymes, and their activity is affected by pH, salt concentration and adsorption of proteins from the blood, but the use of protease-resistant, synthetic peptides can correct problems with activity and proteolysis. Cytotoxicity can also be a problem but is determined by the presence of cationic and hydrophobic moieties and their position within the peptide, so synthetic peptides can be designed with lower toxicity [134]. Different biological modifications have advantages and disadvantages (Table 4), such as stability in vivo, increasing resistance among microbes, degradation by host proteases and non-specific interactions. In general, AMPs are active against a broad spectrum of microorganisms, effective at low doses (reducing toxicity), combat microbes that are resistant to antimicrobial drugs or antibiotics and do not trigger immune responses [135]. AMPs are degraded rapidly in vivo, and synthetic AMPs, which are more resistant to degradation, are expensive to produce [136]. Antibiotics are critical for treating the early stages of an infection to prevent sepsis and for prophylaxis in high-risk patients, but some antibiotics have serious side-effects, including allergic reactions, toxicity and interaction with other drugs, damage to the patient’s microbiome and increasing microbial antibiotic resistance [137].

Examples of the use of biological modifications of surfaces are presented below and in Table 4.

### 5.1. Antimicrobial Peptides (AMPs)

**Figure 12:**
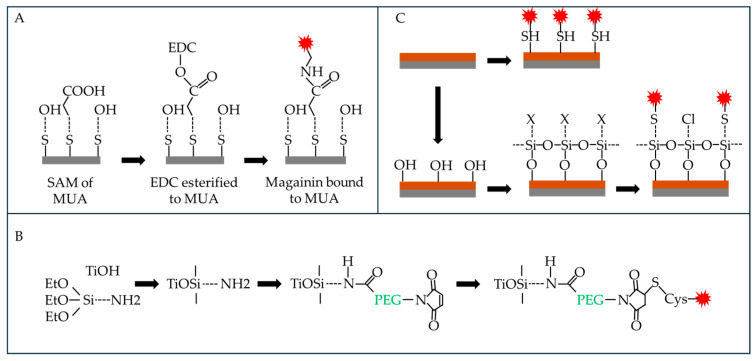
Attachment of AMPs to metal surfaces. (**A**) magainin attached to a SAM of MUA on a gold surface, (**B**) site-specific coupling of LL-37 to titanium, (**C**) attachment of hLf1-11 to titanium by physical adsorption (top) and by silanization and covalent bond with CPTES. EDC: 1-ethyl-3-(3-dimethylaminopropyl) carbodiimide, SAM: self-assembled monolayer. MUA: 11-mercapto-undecanoic acid, PEG: poly(ethylene glycol), CPTES: 3-chloropropyltriethoxysilane, Gray: surface being modified, orange: SAM, red explosion symbol: antimicrobial peptide. Adapted from [139].

Thousands of AMPs have been isolated, mainly from animals, and they kill microbes (Table 1) by forming pores in, or otherwise disrupting, the microbial membrane by inhibiting cell wall, nucleic acid or protein biosynthesis or by blocking the activity of enzymes [138,139]. Fungi such as *C. albicans* have evolved strategies to combat AMPs, including proteases that degrade AMPs, peptides that bind and inhibit them, membrane transporters that pump AMPs out of the fungal cell and increased chitin biosynthesis to counteract AMPs that target chitin synthesis [140]. Methods of attaching AMPs to surfaces include the attachment of magainin via a self-assembled monolayer of 11-mercaptoundecanoic acid (Figure 12A), site-specific attachment of LL-37 to titanium via a PEG spacer (Figure 12B) and attachment of hLf1-11 to titanium by adsorption or by silanization with 3-aminopropyltriethoxysilane (Figure 12C) [139].

#### 5.1.1. Magainins (MAGs)

**Figure 13:**
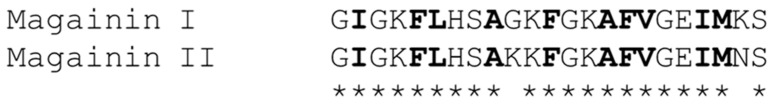
The sequences of magainin I and magainin II were aligned in Clustal Omega [143]. Hydrophobic residues, shown in bold, * identical conserved residues. Adapted from [142].

MAGs were first isolated from the skin of *Xenopus laevis (*Table 1) and are active against several Gram-positive and Gram-negative bacteria as well as *C. albicans* [141]. The sequences of magainin I and magainin II [142] were aligned in Clustal Omega [143]. The two sequences differ in 2 amino acid residues out of 23 (Figure 13). Magainins have hydrophobic (bold) and hydrophilic (plain text) portions, allowing them to interact with the cell membrane and form pores and micelles, thus disrupting the membrane and killing microbes. 11-mercaptoundecanoic acid (MUA) can be assembled into a single layer on surfaces such as gold, the carboxylate group converted to an ester and magainin I attached via its amino groups to the MUA ester group. In experiments with the MAG-modified gold, over half of the adhered *Listeria ivanovii*, *S. aureus* and *Enterococcus faecalis* were killed within 30 min [84,139].

#### 5.1.2. Human Cathelicidin, LL-37

LL-37 is the only cathelicidin AMP found in humans. It kills *Pseudomonas*, *Escherichia*, *Staphylococcus* and *Enterococcus* species. Furthermore, it blocks biofilm formation and adherence and disrupts biofilms of *S. aureus* and *P. aeruginosa*. However, LL-37 is cytotoxic, readily degraded by proteases, expensive to synthesize and less active in vivo. Several organisms, including *S. aureus*, *S. typhimurium* and *Clostridioides difficile*, have been shown to develop resistance after multiple passages in the presence of a sub-lethal dose of LL-37. Attaching LL-37 to a surface helps combat problems with cytotoxicity and proteolytic degradation. When LL-37 was connected to a titanium surface via polyethylene glycol, it actively killed *E. coli*. Some groups have produced synthetic versions of LL-37 with an altered sequence and have reported reduced cytotoxicity and instability, but others have reported the opposite and also a narrower antimicrobial spectrum, reduced activity against biofilms, poorer membrane disruption and lower in vivo activity [85,139,144].

#### 5.1.3. hLF1-11

The AMP hLF1-11 is based on the N-terminus of human lactoferrin and can be attached to the hydroxyl groups created by oxygen plasma treatment of titanium surfaces (Table 1), reducing adhesion and biofilm formation by *Streptococcus sanguinis* and *Lactobacillus sailvarius*. Three attachment methods were tested―silanization and two types of copolymer brush methods. The copolymer brush methods were more effective at reducing bacterial colonization than the simple salinization method, probably because there was a greater density of hLF1-11 [86]. Lactoferrin is believed to affect bacteria by sequestering the iron that bacteria need to grow and replicate, disrupting the membrane by binding to a component of Gram-negative bacterial lipopolysaccharide, and degrading virulence factors of *Shigella* species and *E. coli* in a manner that depends on interaction between lactoferrin and target proteins on the microbial cell surface, including lipid A of Gram-negative bacteria or *S. epidermidis* lipoteichoic acid [145].

#### 5.1.4. Melimine

**Figure 14:**
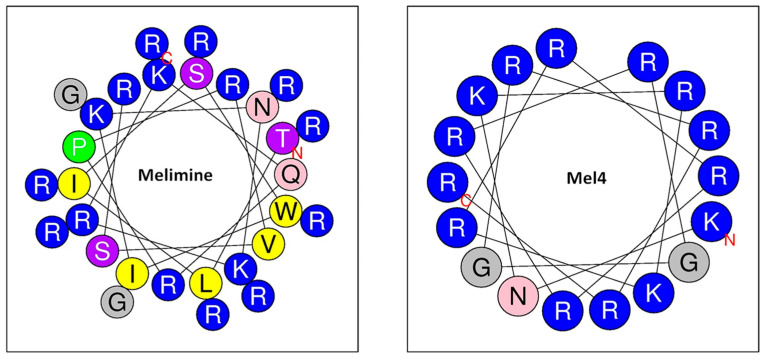
Structure of melimine and Mel4. Blue: positive charge, gray: uncharged, pink: polar, yellow: hydrophobic residues. Reproduced from [147] https://creativecommons.org/licenses/by/4.0/ (accessed on 20 April 2025).

The artificial AMP melimine (Table 1) is made up of fragments of the AMPs mellitin (from honeybee venom) and protamine (from salmon sperm) and damages bacterial membranes, including those of *S. aureus* and *P. aeruginosa*, and can be attached to a surface, where it inhibits adherence of bacteria. Melimine possesses a mix of hydrophobic and polar/charged residues. A tryptophan residue or perhaps leucine or isoleucine residues are believed to be important in the interaction of melimine with lipids of the bacterial cell membrane. The newer Mel4 molecule lacks these residues and is less cytotoxic but permeabilizes cell membranes more slowly than melimine (Figure 14). Melimine was attached to titanium via a silane moiety. First the titanium was treated with 3-aminopropyltriethoxysilane to attach amine groups to the surface. Then, 4-(N-maleimidomethyl) cyclohexane-1-carboxylic 3-sulfo-n-hydroxysuccinimide ester (sulfo-SMCC) was attached to these groups and melamine was attached to the sulfo-SMCC. *S. aureus* colonization was reduced by 84% and that by *P. aeruginosa* by 62% on melimine-covered titanium compared with bare titanium. Melimine alters the architecture of the cell membrane, and repeated rounds of microbial growth in the presence of a sub-inhibitory dose of melimine did not result in resistance to the AMP [87,146].

#### 5.1.5. Cateslytin

Medical devices may be coated with layers of hyaluronic acid, the antimicrobial peptide cateslytin and chitosan (Table 1). When pathogens secrete hyaluronidase, the enzyme degrades the chitosan coating, leading to the release of cateslytin, which inhibits the growth of *C. albicans* and *S. aureus* [88]. Alternatively, devices may be coated with layers of poly-L-lysine and poly-L-glutamic acid in which the antifungal peptide CGA 47–66 has been embedded. The peptide diffuses out of the coating and inhibits *C. albicans* growth by 65% and completely blocks the growth of *Neurospora crassa*. *N. crassa* is not normally considered to be a pathogen, but *Neurospora* spp. have been implicated in several fungal infections, often in patients with COVID-19 infection and/or type 2 diabetes [148,149,150].

### 5.2. Antibiotics

**Figure 15:**
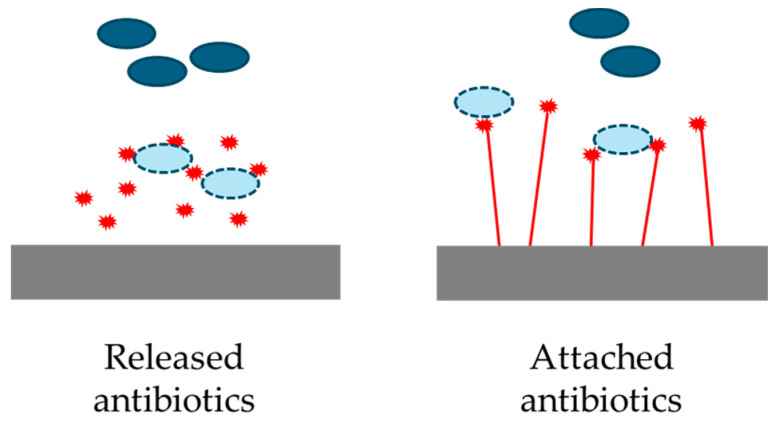
Use of antibiotics to protect surfaces. Gray: surface from which antibiotics are released or to which they are attached, dark blue: live microorganisms, light blue: dead microorganisms, red: explosion symbols: antibiotic. Adapted from [151].

Antibiotics (Table 1) may be attached covalently or by adsorption to various surfaces (Figure 15) [151]. Covalent attachment extends the period over which the antibiotic is effective as it avoids desorption, which could also lead to unwanted toxicity as desorbed antimicrobials accumulate in various tissues. Alternatively, antibiotics may be released from the surface, either at once or over time (leading to possible undesirable toxicity). If attached to the surface, the antibiotic is immobilized and acts locally when microorganisms approach the surface being protected [82]. Antibiotics may be biostatic (blocking growth/replication) or biocidal (killing microorganisms by a range of mechanisms) [152].

#### Gentamicin and Other Antibiotics on Hydroxyapatite Coatings

Gentamicin (Table 1) was originally extracted from the bacterium *Micromonospora purpureais.* It is active against a wide variety of bacteria and does not denature under mild heat treatment. It is therefore preferred for attaching to hydroxyapatite (HA) coatings on metal implants to protect them from biofilm formation [153]. Rabbit tibias were infected with a clinical strain of *S. aureus*, and then steel wires were implanted either with HA but without antibiotics or with gentamicin on HA. In all, 88% of test animals were infected when gentamicin was not used, whereas no test animal was infected where HA-covered wires had gentamicin attached. Biofilm formation on wires and in bones was significantly reduced when gentamicin was used [83]. Another group incorporated antibiotics into carbonated hydroxyapatite on titanium. A wider range of antibiotics could be used since the method was carried out at 37 °C. They crystallized the calcium phosphate and HA and co-precipitated the antibiotics from a solution of calcium and phosphate. Antibiotics with carboxylate groups (cephalothin, carbenicillin and cefamandol) were better incorporated and more slowly released than those without [154].

## 6. Conclusions

All surface modification strategies have advantages and disadvantages. Some modifications cause inflammation, affect wound healing or bone deposition or are toxic to host cells [57,59,61]. Combining techniques may have synergistic effects, e.g., slippery liquid-infused porous surfaces (SLIPSs) [155] repel and prevent the adherence of microbes and can be loaded with hydrophobic drugs. Drugs diffuse slowly and prevent the growth of non-adhered microbes. PEG brushes combined with cerium (IV) oxide and trimanganese tetraoxide may be used to modify the surface of titanium [156]. The PEG component resists the adherence of microbes, while the manganese and cerium ions trigger the production of ROS that damage the membrane and cause cell death, and the trimanganese tetraoxide oxidizes glutathione, countering its antioxidant activities. Poly-N-isopropyl-acrylamide hydrogels form gels above a certain temperature but solutions below it, and the addition of other compounds produces coatings that slough off microorganisms when the temperature is changed [157]. Another approach is to degrade quorum-sensing molecules [158] or the ECM [47]. One group attached enzymes to the anticorrosion agent, polyaniline, and reported that embedded α-amylase reduced marine biofilm formation by 76.5% [159]. Bacteriophages or their proteins can disrupt biofilm [46], and techniques have been developed to attach bacteriophages to gold, glass, cellulose or hydrogels via adsorption, electrostatic interaction or covalent bonds [58].

Given the growing incidence of drug-resistant bacteria and fungi, it is vital to identify effective means of protecting industrial and medical equipment and devices from microbial adhesion and biofilm formation. There are many promising biocidal or biostatic compounds that could be embedded in or attached to surfaces or coatings. However, in addition to the optimization of the surface treatments themselves to prevent adhesion and biofilm formation, the question of the durability of such a treatment and the maintenance of its long-term functionality is crucial. For example, if physical surface structuring or the chemical formation of a brush-like coating is used, such a structure must be able to withstand the conditions under which it is used, i.e., mechanical damage/abrasion during movement or the smoothing of surface structures due to the deposition of substances from liquids as they flow through pipes, for example. Similarly, the antimicrobial substances in the treated surface must retain their effectiveness over a sufficiently long period of time. If they gradually dissolve from the surface or are unstable, the antimicrobial effectiveness of the surface is lost. An important challenge is to produce surfaces that allow adhesion/biofilm formation by beneficial microbes but prevent adhesion and pathogenic biofilms.

## Figures and Tables

**Table 1:** Surface modifications to reduce biofilm formation.

Technique	Species Tested	References
Physical		
Laser treatment	*S. cerevisiae*	[62]
	*S. aureus*	[63]
	*E. coli*	[63]
Dip pen nanolithography	*S. mutans*	[64]
Plasma treatment	*P. aeruginosa*	[57]
	*S. aureus*	[57]
	*E. coli*	[57]
	*S. epidermidis*	[57]
Reactive ion etching	*B. subtilis*	[65]
	*S. aureus*	[65]
Electron beam	*S. aureus*	[66]
	*S. epidermidis*	[66]
	*P. aeruginosa*	[66]
Electron beam melting	*C. albicans*	[67]
	*P. aeruginosa*	[67]
	*S. aureus*	[67]
Physical vapor deposition	*P. fluorescens*	[68]
Chemical		
Polyethylene glycol	*S. epidermidis*	[69]
	*S. aureus*	[69]
	*S. salivarius*	[69]
	*E. coli*	[69]
	*P. aeruginosa*	[69]
	*C. albicans*	[69]
	*C. tropicalis*	[69]
Zwitterions	*C. albicans *	[70]
	* P. aeruginosa *	[71]
Add polyethylene oxide to polyurethane	* C. albicans *	[37]
Add acrylic polymer to zirconia	* S. sanguinis *	[72]
	* P. gingivalis *	[72]
	* F. nucleatum *	[72]
	* C. albicans *	[73]
Fluorosiloxane coating	* S. aureus *	[58]
Add organic compounds to polyurethane	* S. epidermidis *	[74]
Graft polyNaSS onto the surface of titanium	* S. aureus *	[75]
Tether QACs to polysiloxane to coat aluminum	* C. lytica *	[76]
	* N. incerta *	[76]
Paint QACs on titanium or stainless steel	* S. aureus *	[77]
DMPEI on PVC	* E. coli *	[78]
	* S. aureus *	[78]
	* C. albicans *	[78]
Embed silver nanoparticles in titanium	*S. aureus*	[79]
Calcium phosphate coatings	*P. gingivalis*	[80]
Diffusion of Toremifenefrom sol-filled pores in Ti	*C. albicans*	[81]
Attach to roughened titanium via a silane anchor	*S. aureus*	[82]
	*P. aeruginosa*	[82]
Attach gentamicin to hydroxyapatite coating	*S. aureus*	[83]
Biological		
Attach magainin to SAM of MUA on gold	*L. ivanovii*	[84]
	*S. aureus*	[84]
	*E. faecalis*	[84]
Attach LL-37 to titanium via silanized PEG	*E. coli*	[85]
Attach hLf1-11 to surface via silane/copolymer brush	*S. sanguinis*	[86]
	*L. sailvarius*	[86]
Attach melamine to surface via silane	*S. aureus*	[87]
	*P. aeruginosa*	[87]
Embed cateslytin between hyaluronic acid and chitosan	*C. albicans*	[88]
	*S. aureus*	[88]

**Table 2:** Advantages and disadvantages of physical modifications.

Advantages	Refs	Disadvantages	Refs
Surface roughening and altered wettability can be effective against a broad spectrum of microbes	[57,63,66]	In some experiments, the surface roughening effect is species-specific	[67]
Molds can be made of modified surfaces to cast surface patterns in cheaper plastics	[64]	Requires expensive equipment, though cheaper laser techniques can be effective	[91]
Nanopillars and other structures are microbiocidal	[95]		
Nanopillars appear to be biocompatible	[90]		
No need for toxic chemicals	[61]		

**Table 3:** Advantages and disadvantages of chemical modifications.

Advantages	Refs	Disadvantages	Refs
PEG is highly effective and safe to use in vivo	[61]	Ether link can be oxidized in vivo, and attaching other molecules can trigger an immune response	[61]
Easy to manipulate characteristics of hydrogel via altering chemical structure and easy to attach	[61]	Some hydrogels are toxic. First-generation chitosan hydrogels are toxic, but later generations are not	[61]
Zwitterions become highly hydrated, which is effective at reducing microbial adherence. They have good biocompatibility, low toxicity, cause almost no immune reaction, are stable and persist in the body for a long time	[111]	Some zwitterionic polymers are not very biocompatible	[61]
QACs are stable, have low toxicity and are effective at low concentrations. They kill microbes on contact, and some also reduce microbial adhesion	[61]	QACs persist in the environment and can trigger allergic reactions, respiratory problems, reproductive issues and endocrine dysfunction	[112]
Superhydrophobic surfaces are self-cleaning since liquid rolls off the surface	[61]	Some production methods require expensive equipment, cause pollution or require high amounts of energy, are toxic or lead to poor performance (sol–gel)	[113]
Grafting polyNaSS onto polyaryletherketone reduces microbial adhesion and enhances hydrophilicity, protein adsorption, bone repair and biocompatibility	[114]		
A hydrogel containing PAMAM and ketoconazole (KET) had a greater effect on *C. albicans* viability than one containing only KET, possibly because PAMAM enhanced the solubility of KET	[115]	Later-generation cationic dendrimers take a long time to synthesize, are cytotoxic and are quickly cleared from the body	[116]
Dendrimer degradation by proteases is low, resulting in high bioavailability. Effective at low concentrations. Peptide dendrimers are more effective and exhibit higher biodegradability	[117]	Dendrimers are cytotoxic and trigger an immune response when the density of dendrimers is high. Synthesis and purification are difficult, with a high percentage of impurities	[117]

**Table 4:** Advantages and disadvantages of biological modifications.

Advantages	Refs	Disadvantages	Refs
AMPs are broad-spectrum, effective at low dosages, combat microbes that are resistant to antibiotics, do not trigger an immune response, and exhibit low toxicity	[135]	AMP degradation by proteases in vivo leads to low bioavailability. Synthetic AMPs are more resistant to degradation but are expensive to produce	[136]
Antibiotics are critical for treating infection during the early stages to prevent sepsis and for prophylaxis in high-risk patients	[137]	Some antibiotics have serious side-effects, including allergic reactions, toxicity and interaction with other drugs, damage to the patient’s microbiome and increasing microbial antibiotic resistance	[137]

## Data Availability

Not applicable.

## Appendix A

Literature Search Methodology: Relevant literature was identified primarily through PubMed and Google Scholar using the keyword combinations “biofilm”, “surface modification”, “antifouling”, “bacteria” and “fungi”. Other terms included “surface roughness”, “nanopillar”, “nanotopology”, “superhydrophobicity”, “superhydrophilic-ity”, “PEG”, “QAC”, “AMP”, “antibiotic” and other terms referring to various methods of physical modification of surfaces. The source articles with the most recent relevant findings (published within the past 10 years), supplemented by the earliest key reports, were prioritized.
